# Syncopation and the Score

**DOI:** 10.1371/journal.pone.0074692

**Published:** 2013-09-10

**Authors:** Chunyang Song, Andrew J. R. Simpson, Christopher A. Harte, Marcus T. Pearce, Mark B. Sandler

**Affiliations:** Centre for Digital Music, Queen Mary University of London, London, United Kingdom; UNLV, United States of America

## Abstract

The score is a symbolic encoding that describes a piece of music, written according to the conventions of music theory, which must be rendered as sound (e.g., by a performer) before it may be perceived as music by the listener. In this paper we provide a step towards unifying music theory with music perception in terms of the relationship between notated rhythm (i.e., the score) and perceived syncopation. In our experiments we evaluated this relationship by manipulating the score, rendering it as sound and eliciting subjective judgments of syncopation. We used a metronome to provide explicit cues to the prevailing rhythmic structure (as defined in the time signature). Three-bar scores with time signatures of 4/4 and 6/8 were constructed using repeated one-bar rhythm-patterns, with each pattern built from basic half-bar rhythm-components. Our manipulations gave rise to various rhythmic structures, including polyrhythms and rhythms with missing strong- and/or down-beats. Listeners (*N* = 10) were asked to rate the degree of syncopation they perceived in response to a rendering of each score. We observed higher degrees of syncopation in time signatures of 6/8, for polyrhythms, and for rhythms featuring a missing down-beat. We also found that the location of a rhythm-component within the bar has a significant effect on perceived syncopation. Our findings provide new insight into models of syncopation and point the way towards areas in which the models may be improved.

## Introduction

Beat is the underlying periodic percept that human listeners extract from temporal patterns in music [1]. When human listeners infer structure from salient periodicities in beat groupings, the resulting abstract temporal construct is known as *meter* [2], [3], [4]. The primary beat-grouping is marked by a salient event known as the *down-beat*. This primary grouping can then be subdivided at a second level of salience, into *strong-beats* and *weak-beats*. This gives rise to the nested hierarchical structure of meter [2].

**Figure 1:**
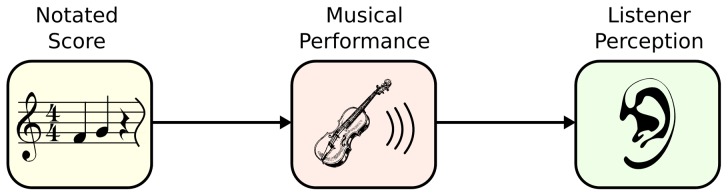
Transformation: *from the score to perception.* Before the notes on the score can be perceived as music by the listener, the score must be rendered (e.g., by a performer) as an acoustic (pressure) signal which varies over time. Therefore, in a psychophysical sense, the score may be defined as the objective correlate of subjective perception. By manipulating the score, we can find out what features of the score correspond to features of perception, in this case syncopation.

The score is a symbolic encoding that describes the set of events comprising a piece of music. Before these notated events can be perceived as music by a listener they must be rendered (e.g. by the performer) as an acoustic pressure signal that varies over time (as illustrated in Fig. 1). Therefore, the rendering process mediates the transformation between the score and the perception. The notation of meter in musical score is known as the *time signature*, which tells the musician how to group beats in time so as to produce the intended perception of meter when the notes are played.

When the prevailing metrical structure inferred by the listener is momentarily contradicted, the resulting percept is known as syncopation [5], [6]. In principle, for such a contradiction to occur it must be assumed that metrical structure for the listener is already established (prior to the syncopated event being experienced), presumably in a way that is equivalent to the time signature written on the score itself. Therefore, whether the notated rhythm in question causes a perception of syncopation must be partly determined by the degree to which a pre-existing metrical structure has been established.

In the score, intended syncopation is typically notated by placing rhythmic *accents* (i.e., salient events) on weak-beats (rather than strong-beats) and by placing rests or tied notes on strong-beats [7]; these are defined as *onset syncopation* in [8] and feature in mathematical models of syncopation [6], [9]–[19]. A common compositional device that leads to syncopation is polyrhythm. Polyrhythm is defined as the simultaneous presentation of two or more periodic rhythms which do not share a common rhythmic grouping [6], [10], [20], often resulting in a sense of competing meters [20], [21].

Syncopation has been generally addressed as rhythmic complexity. Rhythmic complexity has been estimated in terms of experts' ratings [22], rhythm reproduction tasks [18], [23], and rhythm recognition tasks [18]. It has also been shown that syncopated rhythms reduce the accuracy of human beat-tracking [18], [24], [25]. However, to our knowledge, estimation of the subjective strength (magnitude) of syncopation has not previously been attempted. In this paper we investigate the correlates of perceived syncopation directly by asking listeners to provide quantitative estimates of perceived syncopation while we manipulate the rhythmic patterns in the score.

In our experiments we manipulated the temporal structure of the music score and used a metronome to provide explicit cues to the prevailing meter (as defined by the time signature). We constructed three-bar scores, with time signatures of 4/4 and 6/8. The scores contained repeated one-bar rhythm-patterns, where each rhythm-pattern was built from basic half-bar rhythm-components. The metronome preceded the rhythms for one bar and ran concurrent with the rhythms for the final two bars. Our manipulations gave rise to various rhythmic structures, including polyrhythms and rhythms where the down-beat (the first beat in a bar) was missing in some cases. Listeners were asked to rate the degree of syncopation they perceived in response to a rendering of each score.

We test the hypothesis that the following will have a degree of influence on perceived syncopation: i) time signature, ii) whether the down-beat is present or missing, iii) presence of polyrhythms or “monorhythms” (which we will define here as any rhythm pattern which is not polyrhythmic) and finally iv) within-bar location of rhythm components. In our experimental results, we observed higher degrees of perceived syncopation for monorhythms in the time signature of 6/8 than for those in 4/4, for polyrhythms than monorhythms, and for rhythms featuring a missing down-beat and/or strong-beat. We also found that the location of rhythm-components (within a bar) has a significant effect on perceived syncopation. Our findings suggest that current models of syncopation [6], [9]–[15] may have scope for improvement.

## Materials and Methods

### Ethics Statement

Participants were unpaid volunteers and gave informed verbal consent before the experiment. Participants were free to withdraw at any point. Tests were arranged informally and conducted at the convenience of the participants. Written consent was not deemed necessary due to the low (safe) sound pressure levels employed in the test. The experimental protocol (including consent) was approved by the ethics committee of Queen Mary University of London.

### Method overview

Psychophysics applies psychological methods to quantify the relationship between perception and stimulus [26]. A fundamental postulate of psychophysics is that perception should have underlying objective, physical correlates which may be quantified as features of the stimulus. For example, intensity is the objective correlate of loudness (perceived intensity). In this paper we manipulate the score as an objective correlate of perceived syncopation (see Fig. 1).

We asked musicians to give informed ratings of perceived syncopation for renderings of various three-bar scores. The ratings were taken over a fixed, five-point rating scale. In this experiment we required the listeners to judge a large number of rhythms, with a potentially large range of syncopation ratings. The fixed rating scale was intended to provide the minimum complexity in the experimental interface and the maximum efficiency during the procedure; the aim being that listeners would not be hampered by unnecessary precision in the interface and would be able to focus on their immediate perceptual response. We acknowledge that such methods may be prone to minor biases (e.g., range bias, end-point bias [27]) but we argue that such biases are offset by the overall scale of the syncopation continuum and stimuli. In other words, the stimuli we employed ranged between ‘not syncopated’ and ‘highly syncopated’, so in our method we trade finer detail in the data for an efficient method. All listeners used the whole range of the scale (i.e., each listener gave at least one minimum and one maximum rating).

### Participants

We recruited ten trained musicians, nine male and one female, with an average age of 30 years (standard deviation 5.8 years). All participation was voluntary (unpaid). Musical training includes formal performance and theory over a range of instruments, music production and engineering. All participants had trained for an average of 15 years (standard deviation 5). Six of them reported proficiency in multiple instruments. All participants confirmed that they were confident in their understanding and rating of syncopation. All participants reported normal hearing.

### Stimuli

**Figure 2:**
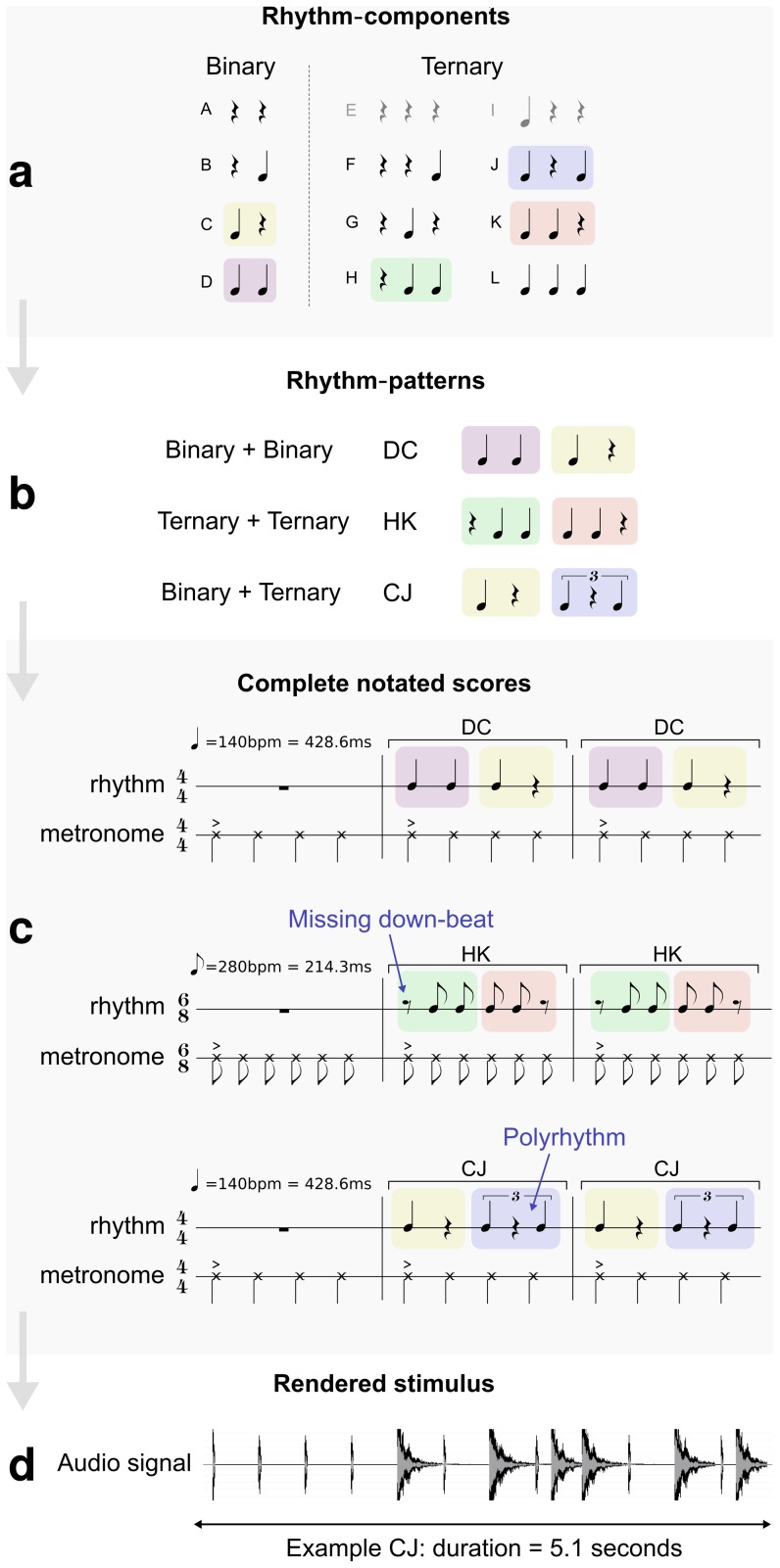
Construction of stimulus. A schematic diagram illustrating the process of generating the stimuli; basic half-bar rhythm-components are paired to create one-bar rhythm-patterns, rhythm-patterns are used to produce a three-bar score (including metronome) and finally the score is rendered as a waveform. **a** shows ‘binary’ and ‘ternary’ grouped rhythm-components. Rhythm-components A, B, F, G and H feature ‘missing down-beats’. **b** shows example rhythm-pattern pairings. **c** shows example scores, including rhythm-patterns featuring missing down-beats and polyrhythms. **d** Shows an example waveform (for rhythm-pattern CJ), rendered using synthesis.

Each score, rendered to produce a single stimulus, was constructed of three bars. The first bar was always metronome alone (either 4/4 or 6/8). The second and third bars were repetitions of a one-bar *rhythm-pattern* constructed from concatenation of two basic, half-bar *rhythm-components*. Figure 2 provides a schematic diagram which illustrates the steps taken when generating the stimuli. First, various half-bar rhythm-components (Fig. 2a) are paired to produce one-bar rhythm-patterns (Fig. 2b). The rhythm-components are categorized as either ‘binary’ (two notes) or ‘ternary’ (three notes). Next, the rhythm-patterns are concatenated and a metronome is added to produce the final score (Fig. 2c). Finally, the stimulus is rendered to produce the acoustic waveform (Fig. 2d) which is ultimately heard by the listener. Rhythms were played concurrently with the metronome (following the single bar of introductory metronome – see Fig. 2c).

Figure 2a shows the ten half-bar rhythm-component notations (A-L) from which concatenated whole-bar pairs were produced in all possible combinations. These base rhythm-components include notations featuring rhythmic structures that are anticipated to result in syncopation: missing down-beats, off-beat notes and polyrhythms when presented in relation to a metronome. Example rhythm-pattern pairings are given in Figure 2b. Rhythm-patterns composed of a given pair of rhythm-components were presented separately in both forward and reverse order (e.g., CJ and JC). By comparing such pairs, we are able to investigate the effect of location (e.g., of missing strong-beats) within the bar.

Scores for example stimuli, including metronome, are given in Figure 2c. There were 99 unique pairs, after excluding redundant patterns E and I, which were replaced with A and C respectively (which are equivalent in 4/4). The time signature was set to 6/8 for all combinations of two ternary rhythm-components and 4/4 for the rest.

The stimuli were rendered (synthesized) at a sampling rate of 44.1 kHz 16-bit using MIDI sequencing (see Fig. 2d for an example waveform). A percussive snare drum sample was used for the musical rhythm and a “cow-bell” sample was used for the metronome. The snare drum sample was approximately 700 ms in duration, with approximately 7 ms attack, 130 ms sustain and 450 ms decay. The metronome sample was relatively impulsive and of approximately 20 ms duration. The metronome was dynamically accented on the first beat of the bar and was also accented in pitch; the fundamental frequency of the accented note was 940 Hz and the remaining notes were of 680 Hz. Thus, our metrical cue (metronome) was clearly differentiable (by timbre and pitch) from the overlaid drum rhythm. By accenting the first beat of metronome in 6/8, we do not explicitly rule out a 3/4 grouping of beats. The tempo of the metronome was set to 140 beats per minute (BPM) for all patterns in a time signature of 4/4 and 280 BPM for those in 6/8. This corresponds to an interval of 428.6 ms per quarter-note in both time signatures. In 4/4 the metronome beat quarter-notes at this interval and in 6/8 it beat eighth notes (i.e., an interval of 214.3 ms per beat). Hence, in 4/4 stimuli that contained polyrhythmic components, the interval between triplet quarter-notes was 285.7 ms. The resulting stimuli durations (per trial) were 5.1 seconds in 4/4 (i.e., three bars of four quarter-note beats) and 3.9 seconds in 6/8 (i.e., three bars of six eighth-note beats).

### Procedure

Stimuli were presented individually and at the instigation of the listener. All stimuli were presented within a single block. For each trial the listener gave a rating between zero and four, where zero indicated *no syncopation* and four indicated *maximum syncopation*. The listener was free to listen to each pattern repeatedly before giving their rating. The stimuli were presented in randomized order (i.e., a different order for each listener). Before the experimental session, the listeners heard a broad range of example stimuli and were given a practice run (the resulting data was discarded). Each participant was free to adjust the sound level at any time so as to be comfortable. All presentation was diotic (same in both ears). Tests were completed in approximately 30–50 minutes. Listeners were encouraged to take breaks during the session. Data and materials are available on request to the corresponding author.

## Results

**Figure 3:**
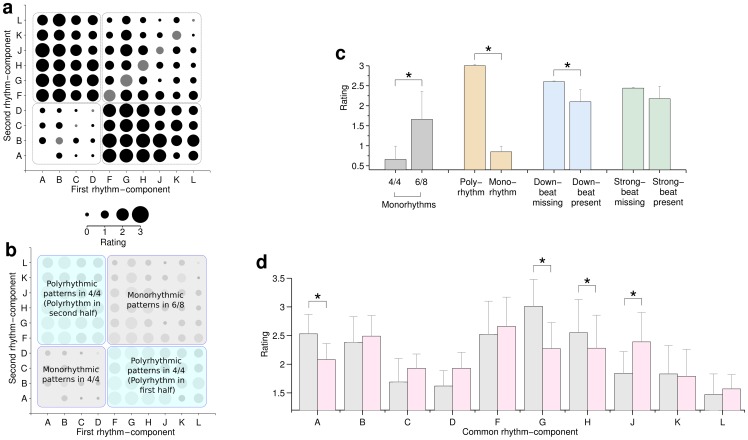
Syncopation by rhythm-component. a Matrix showing group mean syncopation ratings for rhythm-patterns which may be indexed as follows: the upper triangle of the matrix refers to rhythm-patterns where the horizontal axis denotes the first rhythm-component of the rhythm-pattern, and where the vertical axis denotes the second rhythm-component. For the lower triangle of the matrix the reverse is true. This provides a general way to compare the mean ratings between the two orders of presentation for any given pair of rhythm-components. Same rhythm-component pairs (e.g., BB) are shown in grey. Note that the pair AA is excluded because it represents a full bar of rests. **b** shows a ‘map’ of the matrix shown in panel **a**, broken down into regions corresponding to score features: polyrhythmic and monorhythmic patterns in both 4/4 and 6/8. This map illustrates how the data is categorized in the subsequent analyses. **c** shows group mean and 95% confidence intervals for pooled ratings, averaged for each listener, composed (selectively) for comparison of ratings for all stimuli categorized within the following paired conditions: monorhythms in 4/4 versus those in 6/8 (see map in panel **b**), polyrhythms versus monorhythms, down-beat missing versus down-beat present, strong-beat missing versus strong-beat present. * denotes significance (*p*<0.05, *Wilcoxon Signed-Rank Test, uncorrected*). **d** plots mean and 95% confidence intervals for ratings pooled by rhythm-component; For each distribution, all ratings for rhythm-patterns featuring each respective rhythm-component were selected and separated into groups by location of the rhythm-component within the rhythm-pattern (e.g., AB + AC + AD… versus BA + CA + DA….). * denotes significance (*p*<0.05, *Wilcoxon Signed-Rank Test, uncorrected*).

Figure 3a broadly summarizes the syncopation ratings in a matrix representation of the group mean ratings for each rhythm pattern. The horizontal axis shows the *first* rhythm-component of the respective rhythm-pattern, and the vertical axis shows the *second* rhythm-component. Therefore, the upper-left triangular area of the matrix corresponds to the opposite pair-wise ordering of rhythm-components within the same rhythm-pattern to those in the lower-right triangular area of the matrix. Figure 3b provides a ‘map’ – corresponding to Fig. 3a - which illustrates grouping of the ratings for subsequent analyses. Fig. 3c and 3d show various selective groupings of the ratings data (across all listeners), where the data (*N* = 10 listeners) were selected to test the following hypotheses:

### 6/8 is more syncopated than 4/4

For each listener, all ratings were separately pooled and averaged for all stimuli featuring time signatures of 4/4 and 6/8. This gives a pair of ratings distributions which may be compared to see whether either time signature was more or less highly rated (for syncopation). Fig. 3c shows that 6/8 is more highly rated than 4/4 (W = 1, Z = –2.55, *p*<0.01, r = 0.81), *Wilcoxon Signed-Rank Test*).

### Polyrhythms are more syncopated

Next, for each listener all ratings were separately pooled and averaged for all stimuli that constituted a polyrhythm (i.e., in 4/4 – see Fig. 3b) and all stimuli that did not. The resulting ratings distributions are likewise compared to establish the existence of significant differences that may indicate a pre-disposition of polyrhythms to result in the perception of syncopation. Fig. 3c shows that polyrhythms are much more highly rated than monorhythms (W = 55, Z = 2.8, *p*<0.01, r = 0.89, *Wilcoxon Signed-Rank Test*).

### Missing down-beats result in syncopation

For each listener, ratings for all rhythm-patterns featuring ‘missing down-beats’ were pooled and averaged. The same pooled averages were calculated for rhythm-patterns not containing missing down-beats. The resulting group ratings distributions are compared in Fig. 3c and show that rhythm-patterns featuring missing down-beats are more highly syncopated than those not featuring missing down-beats (W = 54, Z = 2.7, *p*<0.01, r = 0.85, *Wilcoxon Signed-Rank Test*). A similar analysis was performed for all pairs featuring missing strong-beats, with a similar (albeit not significant) outcome (*p>0.05*, *Wilcoxon Signed-Rank Test*).

### Switching component order affected syncopation

In order to investigate the effect of location of each rhythm-component within the rhythm-pattern, the ratings resulting from each of the two possible orders were compared. Where certain rhythm-components are associated with high degrees of syncopation (e.g., rhythm-components which feature a missing down-beat), this allows us to observe the effect of location within the rhythm-pattern (bar). For each listener, ratings for all rhythm-patterns featuring a given rhythm-component were pooled and averaged for both possible locations of a given rhythm-component (within the rhythm-pattern). The group mean and 95% confidence intervals for the resulting distributions are plotted in Fig. 3d. Only rhythm-patterns featuring rhythm-components A (W = 34.5, Z = 2.31, *p*<0.05, r = 0.73), G (W = 44, Z = 2.57, *p*<0.05, r = 0.81), H (W = 41, Z = 2.15, *p*<0.05, r = 0.68) and J (W = 0, Z = –2.67, *p*<0.05, r = 0.85) showed significant differences (*Wilcoxon Signed-Rank Test* - *uncorrected*) which held regardless of the other rhythm-components within the various rhythm-patterns. The average ratings were larger when A, G and H were in the first half of the bar, but the opposite was true for J. The overall shape of the graph is consistent with the comparison of missing down-beats shown in Fig. 3c, in that rhythm-patterns featuring rhythm-components A, B, F, G and H show higher mean syncopation ratings.

**Figure 4:**
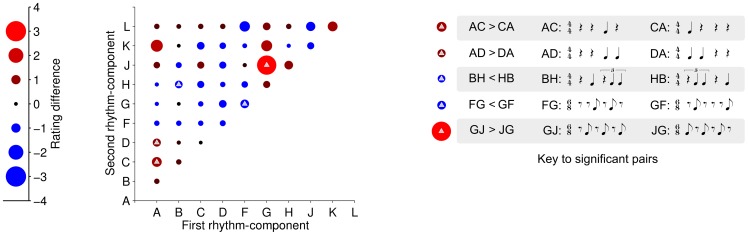
Pair-wise changes in ratings when rhythm-component order was switched. This figure plots (for each rhythm-pattern) the change in group mean rating caused by switching the rhythm-component order (i.e., this is equivalent to a subtraction of the lower-triangle ratings of Fig. 3a from the upper-triangle ratings of Fig. 3a). Triangles denote significance (*p*<0.05, *Wilcoxon Signed-Rank Test, uncorrected*). Interestingly, the significant changes (when order was switched) correspond to *missing down-beat* rhythm-patterns. The right-hand key shows the notations for each pair of rhythm-patterns that reached significance.

In order to find out exactly which rhythm-patterns were sensitive to location of the rhythm-components, the analysis was refined to focus on the pair-wise comparison of ratings for each rhythm-pattern between the two possible orders of the rhythm-components. Figure 4 shows a matrix plot of the difference in group mean rating for each rhythm-pattern, caused by change in the rhythm-component order (i.e., within the bar). Significant changes in rating are indicated with overlaid triangles (*p*<0.05, *Wilcoxon Signed-Rank Test*, *uncorrected*). Rhythm-components which significantly changed when the rhythm-component order was switched were: AC (W = 28, Z = 2.56, *p*<0.05, r = 0.81), AD (W = 15, Z = 2.21, *p*<0.05, r = 0.7), BH (W = 0, Z = –2.21, *p*<0.05, r = 0.69), FG (W = 0, Z = –2.22, *p*<0.05, r = 0.7), GJ (W = 34, Z = 2.28, *p*<0.05, r = 0.72) – see Key of Fig. 4. Again, significant changes occur for rhythm-patterns featuring rhythm-components A, B, F, G, H – all of which feature missing down-beats. In other words, rhythm-components resulting in missing down-beats contribute significantly more to the perception of syncopation than the same rhythm-components in the second half of the bar (rhythm-pattern).

## Conclusions and Discussion

In this paper we have shown that there is more potential for syncopation in 6/8, in polyrhythms and in rhythms featuring a missing down-beat. We have also shown that the location of rhythm-components that give rise to syncopation is critical to its perceived degree. These results demonstrate that syncopation cannot simply be predicted (i.e., in a model) by summation of ‘syncopation values’ calculated for individual notes according to the relationship between each note and the assumed metrical structure. We also identify three questions for further investigation: i) is syncopation tempo-dependent? ii) why do the 4/4 monorhythm patterns exhibit lower syncopation levels than monorhythms in 6/8? iii) do listeners re-interpret the meter of a given rhythm pattern in order to reduce the level of perceived syncopation?

### 4/4 versus 6/8

We employ the standard terminology for meters (i.e., time signatures) in Western music [4]; the terms *duple* and *triple* to refer to two- and three-beat bars respectively, and the terms *simple* and *compound* to refer to the binary and ternary subdivision of beats in a bar. Here, we investigated the signatures 4/4, which is *simple-duple* meter (i.e. two groups of two quarter-notes), and 6/8 which is *compound-duple* meter (two groups of three eighth-notes).

6/8 monorhythmic patterns were rated as more syncopated than those in 4/4 (Fig. 3c). There are several potential explanations for this observation: First, given that a time signature must be rendered (or performed) according to a specified *tempo*, a major difference between the stimuli in these two time signatures is their speed. The beat rate in the 6/8 stimuli was twice as fast as those in 4/4 because eighth-notes are half as long as quarter-notes and the tempi were chosen to maintain the same duration for quarter-notes in both.

It has been shown that tempo influences various aspects of music perception, such as rhythm recognition [21], pitch perception [28], music preference [29] and perception of emotion in music [30]. In particular, the ability to discriminate differences between rhythms [21], perception of meter from polyrhythms [20], [31] and production of rhythmic timing [32] all appear to be influenced by tempo. Therefore, we expect that tempo may affect the perceived syncopation and hence may explain the higher ratings in 6/8 than in 4/4.

Another possible reason for higher ratings in 6/8 than 4/4 may be that the rhythmic structure of 4/4 is inherently less ambiguous - 4/4 is simple-duple meter (duple subdivision of duple) and 6/8 is compound-duple meter (triple subdivision of duple). Several studies have shown that listeners of all ages naturally show bias towards processing (and preference for) rhythms that incorporate binary rather than ternary metrical subdivisions [2], [23], [33], [34]. Indeed, it has been shown that the accuracy of rhythm reproduction in binary subdivisions of beat is higher than ternary subdivisions [33]; people are inclined to tap on the binary subdivisions to isochronous auditory sequences when they are asked to tap at a fast rate [35]; also, both adults and infants react more quickly and accurately to the alterations in pitch, melody and harmony in binary meter than in triple meter [34], [36].

Syncopation has been associated with human metrical processing [18], [22], [24], [25], and metrical processing has also been related to time signature [2], [23], [33], [34], [36]. Our finding, of 6/8 monorhythms being perceived as more syncopated than those in 4/4, suggests that time signature and perceived syncopation are inherently related and hence may explain the previously reported relationship between metrical processing and time signature.

### Missing down-beats

Syncopation models predict that missing strong-beats (the absence of events at strong metrical positions) result in syncopation [9]. The models also predict that a missing down-beat (the first beat of the bar) generates a higher degree of syncopation than a missing strong-beat in a lower metrical level (e.g., the third quarter-note in 4/4 or the fourth eighth-note in 6/8) result in syncopation.

In general, our results agree with the modeling predictions; the patterns with missing down-beats tend to have higher average ratings (Fig. 3c). This is also clear in Fig. 3d, which shows that rhythms starting with a rest (components A, B, F, G and H) contribute to higher average ratings, while patterns including components C, D, K or L have relatively low average ratings (these do not start with a rest).

The latter modeling prediction, that missing down-beats will have a higher degree of syncopation than equivalent missing strong-beats, is partially supported in Fig. 3d: Rhythm-patterns *beginning* with rhythm-components A, G and H (which contain missing down-beats) have higher average ratings than those with A, G or H respectively in the second half (Fig. 3d). The pairwise comparisons (in Fig. 4) for pairs AC/CA, AD/DA and GJ/JG also support this.

### Possible interpretation of 6/8 as 3/4

In Fig. 4 we observe significant difference in syncopation ratings for the 6/8 patterns FG/GF and GJ/JG depending on component order. We might expect to see this for GJ/JG because GJ has a missing down-beat whereas JG does not. Note, however, that this does not explain why other similar 6/8 patterns do not show an equivalent significant difference. In contrast, FG and GF both exhibit a missing down-beat so it is interesting that there should be a significant difference (due to switching order) in this case and prompts further explanation. In listening tests, Povel and Essens [23] found that, given a choice, listeners select the meter which minimizes metrical contradiction (i.e., syncopation). Looking at the rhythm patterns in question (notated in Fig. 4), we can see that for FG and JG, all the notes fall on strong-beats in 3/4 (i.e., eighth-note positions 1, 3 and 5 in 6/8) whereas in GF and GJ, this is not the case. Indeed, using the clock model of Povel and Essens [23], patterns FG and JG are strongly predicted to be interpreted as 3/4 time whereas GF and GJ would be predicted as 6/8. It is possible therefore that the listeners are interpreting some 6/8 patterns as 3/4, which would thus reduce the anticipated level of syncopation. The clock model also makes similar predictions with regards to the results shown in Fig. 3d. The ternary components G, H and J show significant differences according to their location in the bar where other ternary components do not. The component order corresponding to low syncopation ratings in these cases may be explained as a result of listeners interpreting the meter as 3/4. Such metrical interpretation is broadly consistent with the findings of Hannon *et al.* [37], who showed that when judging meter, listeners were more likely to choose 6/8 when the tempo the was fast but more likely to choose 3/4 when the tempo was slow.

### Polyrhythms

Polyrhythms were rated as more syncopated than monorhythms (Fig. 3c). In music psychology, polyrhythms are usually dealt with as a separate concept to syncopation [9], [4]. However, if we accept the definition of syncopation as being a contradiction to the prevailing meter, then the introduction of a competing meter (i.e., within a polyrhythm) would clearly also give rise to this phenomenon. The fact that we found polyrhythms to be more syncopated than monorhythms suggests that the challenge to the prevailing meter, from a counter meter, is more substantial than that caused by emphasizing weak-beats over strong-beats in monorhythms.

In Fig. 4, one pattern containing a polyrhythm, BH/HB, shows significant difference when the order of rhythm components is switched. Both components of BH/HB are missing the strong-beat yet HB was rated as significantly more syncopated than BH. This may be explained by the fact that component B is a monorhythm in 4/4 but H is a polyrhythm in that meter. When H is placed in the first half of the pattern it is a polyrhythm that has a missing down-beat, which implies that the syncopation is compounded in this case.

### Limitations of previous models of syncopation

Previous models of syncopation can be categorized into hierarchical models [9], [11], [12], [13], [15], and off-beat models [6], [10], [14]. In hierarchical models, weights, corresponding to the hierarchical metrical structure [2], [38], are applied to notes appearing in ‘syncopated positions’. Taking Longuet-Higgins and Lee's classic model (LHL) for syncopation [9] as an example, weights are applied to different levels of the metrical hierarchy. The model works by finding strong-beat/weak-beat pairs with an event in the weak position but a rest or a tied note in the strong position. The syncopation value for each pair is calculated as the difference of their weights. These local syncopation values are summed to give the global score for a given rhythm pattern.

In contrast, off-beat models focus on off-beat notes, either in terms of note onsets classified as off-beat [10], [13] or the distances of note onset to beat position [6]. A good example of the distance approach is the weighted note-to-beat distance (WNBD) measure [6]. In this model, the syncopation value for a specific note is considered inversely proportional to its distance from the nearest strong-beat position. Crucially, these models consider syncopation of a certain note to be independent of other notes.

Our results indicate that the ‘summation of local scores’ rule employed in previous models is valid to a limited extent. These models can capture features that are expected to give rise to syncopation. For example, rhythm-components B, F, G contribute to high ratings, and are also predicted to cause syncopation by the models because they start with a rest and have one note in a weaker position after the rest. Conversely, the models also capture the finding (from our data) that pairs containing rhythm-components C, D, K or L have relatively low average ratings. However, the models do not appear to capture other features of our data. We have demonstrated that switching the order of rhythm-components within the bar can affect syncopation (Fig. 4). This finding directly contradicts models such as the WNBD [6]. The limitation in such models is the focus on calculating the distance of individual note to the nearest strong-beat and, in particular, this strategy does not consider the location of the notes within the bar. For example, any pair of rhythm-patterns that have the same components (e.g., GJ and JG) produce the same syncopation value in the WNBD model because the distance of each note to its nearest strong-beat remains unchanged after switching the order of rhythm-components; our data shows that syncopation is different in each order.

In many cases, rhythm-patterns that share a common component are predicted to be equally syncopated by the models but our data show different degrees of syncopation. For example, in the LHL model both component J and K carry zero syncopation and so the total syncopation predicted for rhythm-pattern JG will be equivalent to that predicted for KG. However, our data shows that (on average) KG is rated as being more syncopated than JG.

Future work should include extension of the experimental methodology to alternative stimuli (e.g., control for the effect of tempo, use of non-percussive and/or pitched sounds) and modeling that attempts to capture polyrhythms as well as the time-dependent nature of metrical structure formation and contradiction. This modeling should also account for listeners’ apparent bias towards selection of optimal metrical structures - metrical structures which explain the observed pattern of notes with the least degree of contradiction (syncopation).
